# Mapping of the internal structure of human habenula with *ex vivo* MRI at 7T

**DOI:** 10.3389/fnhum.2013.00878

**Published:** 2013-12-23

**Authors:** Barbara Strotmann, Carsten Kögler, Pierre-Louis Bazin, Marcel Weiss, Arno Villringer, Robert Turner

**Affiliations:** ^1^Neurophysics, Max Planck Institute for Human Cognitive and Brain SciencesLeipzig, Germany; ^2^Cognitive Neurology, Max Planck Institute for Human Cognitive and Brain SciencesLeipzig, Germany

**Keywords:** human habenula, habenular nuclei, lateral habenula, medial habenula, *ex vivo*, MRI, 7T

## Abstract

The habenula is a small but important nucleus located next to the third ventricle in front of the pineal body. It helps to control the human reward system and is considered to play a key role in emotion, showing increased activation in major depressive disorders. Its dysfunction may underlie several neurological and psychiatric disorders. It is now possible to visualize the habenula and its anatomical subdivisions—medial habenula (MHB) and lateral habenula (LHB)—using MR techniques. The aim of this study was to further differentiate substructures within human lateral habenula (LHB) using *ex vivo* ultra-high field MR structural imaging, distinguishing between a medial part (m-LHB) and a lateral part (l-LHB). High resolution T1w images with 0.3-mm isotropic resolution and T2^*^w images with 60-micrometer isotropic resolution were acquired on a 7T MR scanner and quantitative maps of T1 and T2^*^ were calculated. Cluster analysis of image intensity was performed using the Fuzzy and Noise Tolerant Adaptive Segmentation Method (FANTASM) tool. Ultra-high resolution structural MRI of *ex vivo* brain tissue at 7T provided sufficient SNR and contrast to discriminate the medial and lateral habenular nuclei. Heterogeneity was observed in the lateral habenula (LHB) nuclei, with clear distinctions between lateral and medial parts (m-LHB, l-LHB) and with the neighboring medial habenula (MHB). Clustering analysis based on the T1 and T2^*^ maps strongly showed 4–6 clusters as subcomponents of lateral and medial habenula.

## Introduction

**Figure 1:**
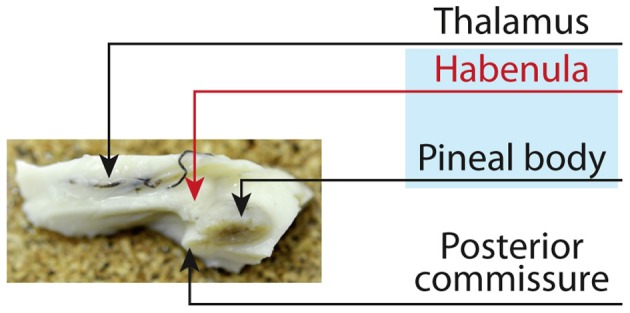
**The Habenula, *the small rein*, sits next to the third ventricle above the thalamus prior to the posterior commissure**. Together with the pineal body the habenula is regarded as the epithalamus. The habenular commissure connects the habenula on both hemispheres and forms a trigone in front of the posterior commissure [published in Strotmann et al. (2013)].

The habenula (HB) is a small but important nucleus located next to the third ventricle in front of the pineal body (Figure 1). It is considered to play a key role in controlling emotion (Hikosaka et al., 2008; Hikosaka, 2010), and its dysfunction may underlie several neurological and psychiatric disorders: Overactivation is associated with depression (Morris et al., 1999; Sartorius and Henn, 2007). The habenula is divided into a medial and lateral habenula based on histological investigations (Herkenham and Nauta, 1977, 1979), mainly receiving inputs from limbic and basal ganglia forebrain structures through the stria medullaris and projecting via fasciculus retroflexus to dopaminergic, serotonergic, and noradrenergic midbrain areas.

Herkenham and Nauta (1977) proposed a heterogeneous architecture of the lateral habenula that included a medial part (m-LHB) associated with the limbic system and a lateral part (l-LHB) associated with the motor system. Diversity within lateral habenula has also been reported by Iwahori (1977) and Andres et al. (1999). Histology of the human brain has shown the division into a medial and lateral habenula (Riley, 1943; Ranft et al., 2010). Alterations of habenula volume associated with a cell loss have been reported in studies investigating patients with major depressive disorder both *in vivo* (Savitz et al., 2011) and *ex vivo* (Ranft et al., 2010).

Few studies have investigated the anatomical complexity of the habenula. However, these studies have mainly focused on the histology of the rat and cat brain. At standard field strengths (=3T) MRI of the habenula shows few internal details. The habenula shares properties of both gray and white matter that only become visible at a high enough field strength and spatial resolution.

It has recently been shown that human habenula and its subdivisions—medial habenula (MHB) and lateral habenula (LHB)—can now be clearly visualized using MRI (Strotmann et al., 2013). This study aimed to use *ex vivo* ultra-high field MR structural imaging to further differentiate between volumetrically different subregions within the lateral habenula (LHB), and also to distinguish between m-LHB and l-LHB.

## Materials and methods

### Data acquisition

**Figure 2:**
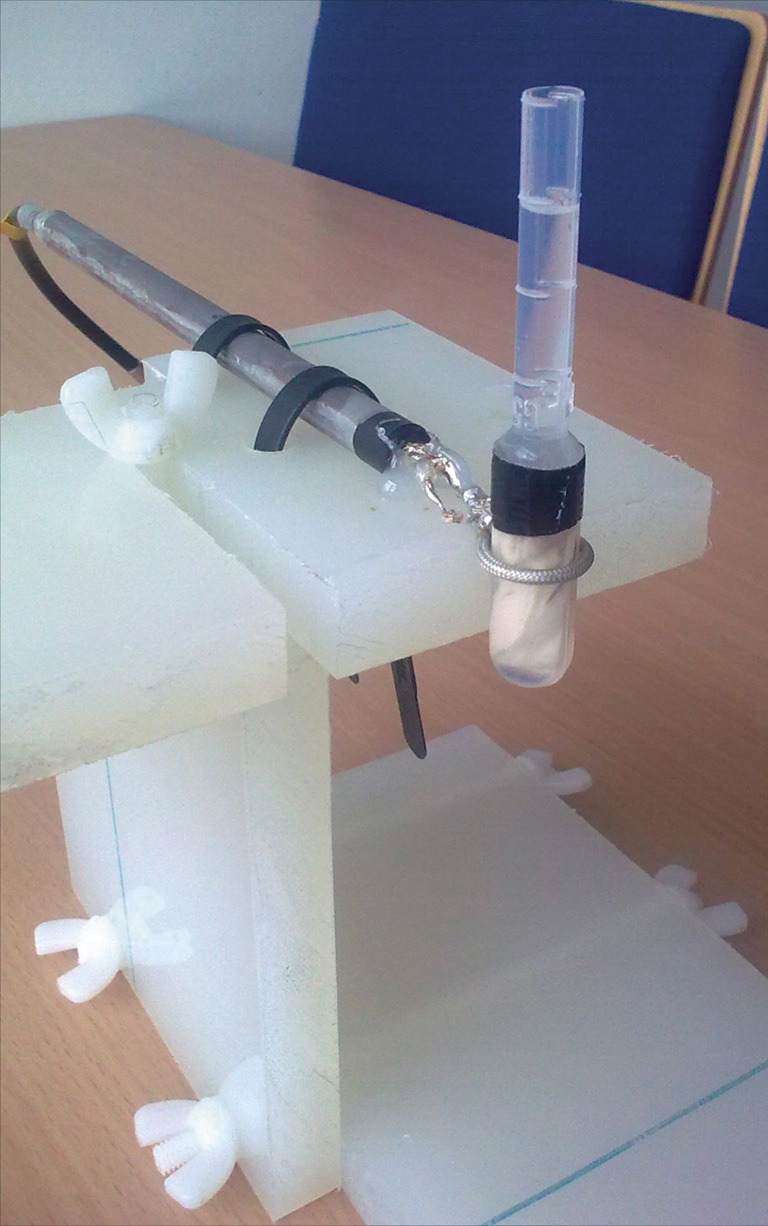
**Miniature loop coil with post mortem sample**.

MRI experiments were performed on a 7T whole-body MR scanner (MAGNETOM 7T, Siemens Healthcare, Erlangen, Germany) with a custom-built miniature loop coil for post mortem brain samples (Figure 2). To this end, one post mortem brain was fixed in 4% formalin within 24 h after death (female, 65 years old, cardiac failure). Formalin fixation of brain tissue decreases relaxation parameters (Tovi and Ericsson, 1992; Dawe et al., 2009), which needs to be considered when choosing parameters for MR image acquisition.

These post mortem results are consistent with *in vivo* data obtained from volunteer human subjects (Strotmann et al., 2013), given the poorer spatial resolution available *in vivo*.

**Figure 3:**
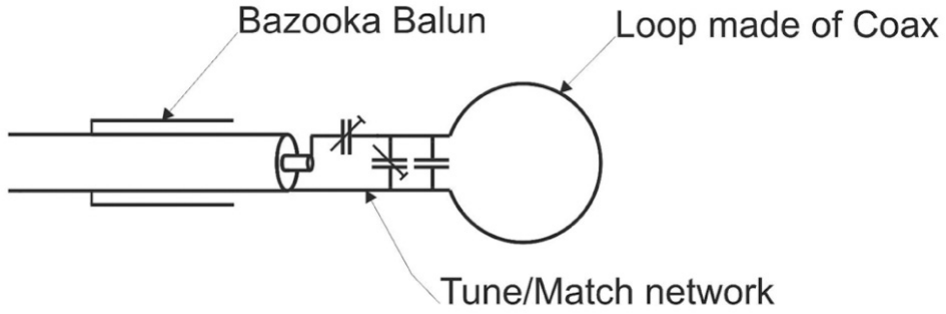
**Custom-built miniature single loop coil**.

The coil consisted of a single loop made of tin-plated semi-rigid coax (coax diameter 2.2 mm) with a 13-mm inner diameter (Figure 3). The sample was placed in a plastic pipette centered in the loop. The cylindrical shape of the pipette avoided unwanted B_0_ distortions.

The loop was tuned to 297.2 MHz and matched to 50 Ohm with variable capacitors (Thin-Trim 9402 series, Johanson Manufacturing, USA) that allowed the coil to be adjusted to different loads. Fixed value capacitors were connected in parallel to compensate for the small quality factor of the trimmer capacitors. A bazooka balun was used to suppress unwanted cable waves. The supporting frame was made of polypropylene, a low loss dielectric material. A separate Transmit/Receive-Switch (Stark Contrast, Erlangen, Germany) was used for interfacing with the scanner.

We acquired high resolution maps of T_1_ with 0.3-mm isotropic resolution using an MP2RAGE sequence (Marques et al., 2010) (*TR* = 3000 ms, *TE* = 2.61 ms, TI1 = 150 ms, TI2 = 900 ms, 4 averages). For T^*^_2_ contrast we obtained data with 60-μm isotropic resolution using a 3D FLASH (Fast Low Angle Shot) multi-echo gradient echo sequence (GRE) with the following parameters (*TR* = 54 ms, 288 slices, flip angle = 25°, 9 averages). Echo times (TE) used were 10, 20, 29.9, 39.8, and 54 ms.

### Image analysis

MRI images and brain sections were compared with macroanatomical landmarks (Mai et al., 2008), and FSL (FMRIB Software Library, University of Oxford 2006, http://www.fmrib.ox.ac.uk/fsl) and the software package MIPAV [Medical Image Processing, Analyzing and Visualization (http://mipav.cit.nih.gov/, version 5.4.4)] from the National Institute of Health (NIH) with its plug-ins were used for further analysis of the data. Given the high degree of inter-subject variability and asymmetry of the structure, defining subcompartments of the habenula properly required an accurate delineation of the complete tissue for the data set. Boundaries were defined based on the structural identification of anatomical features (Riley, 1943; Mai et al., 2008).

The use of different contrasts provided additional information regarding the properties of habenular tissue. For this purpose the MRI parameters T_1_ (using the MP2RAGE sequence) and T^*^_2_ (using a GRE sequence) were quantitatively mapped.

**Figure 4:**
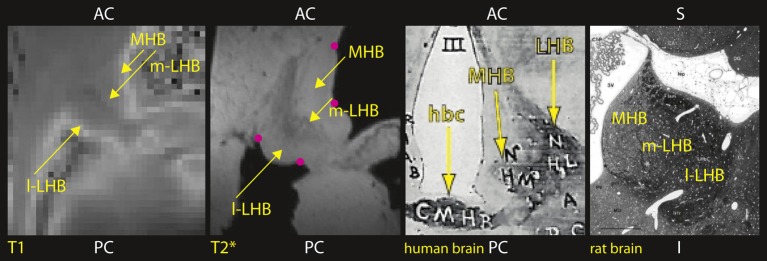
Human Habenula *ex vivo* (axial view, from left to right): Maps of T1 and T2^*^ show distinct habenular subcompartments: lateral habenula (LHB) with a lateral part (l-LHB) and a medial part (m-LHB) and medial habenula (MHB). In comparison, histological stain section of human (axial view) and rat brain (Andres et al., 1999) (coronal view). Anatomical landmarks used for registration as indicated by pink dots.

Initially, we selected four anatomical landmarks (on the brain tissue surface close to the habenula, as indicated in Figure 4) to register the T_1_ map to the higher resolution T^*^_2_ image, and minimized the distance between landmarks selected in the two images with a least square algorithm (Arun et al., 1987). This approximation was further improved by an optimized automated registration algorithm (Jenkinson and Smith, 2001).

To improve the accuracy of the registration, the inversion recovery image with the longest inversion time (INV2) of the MP2RAGE data was co-registered to the T^*^_2_ image using the same registration procedure and the results were compared. The inversion recovery image with the second inversion time shows much better contrast-to-noise ratio across different tissue types. Therefore, we registered the T_1_ map to the results of the INV2 to the T^*^_2_ data.

As extraneous tissue might contaminate the clustering results, we created a habenula mask based on the higher resolution T^*^_2_ image, used both modalities to cross-check the mask and applied to both contrasts. To distinguish between subcompartments within habenula, images were segmented by the Fuzzy and Noise Tolerant Adaptive Segmentation Method (FANTASM) tool (Pham and Prince, 1999) of the TOADS-CRUISE plugins for MIPAV (http://www.nitrc.org/projects/toads-cruise/). For a given input number of tissue classes, this tool classifies each pixel of the images as belonging to a particular class, and further estimates inhomogeneity, attributing a membership value from 0 to 1 for each class (Bazin et al., 2007). After the number of clusters has been selected, the intensity centroids, gain fields, and membership values are calculated. We performed this analysis for *k* = 2, 3, 4, 5, 6, 7 clusters within the habenula.

## Results

Figure 4 shows, in axial sections through the habenula, computed parameter maps of the relaxation times T_1_ and T^*^_2_. Lateral habenula (LHB) and medial habenula (MHB) are clearly visible. The habenula can be visualized on both maps and therefore shows signal characteristics of both gray and white matter. Subdivisions of lateral habenula, medial (m-LHB), and lateral (l-LHB) can be distinguished. The medial part (m-LHB) shows a higher contrast from the surrounding brain tissue and differs from l-LHB and medial habenula (MHB), and we compared to the histology of human and rat brain (Riley, 1943; Andres et al., 1999).

**Figure 5:**
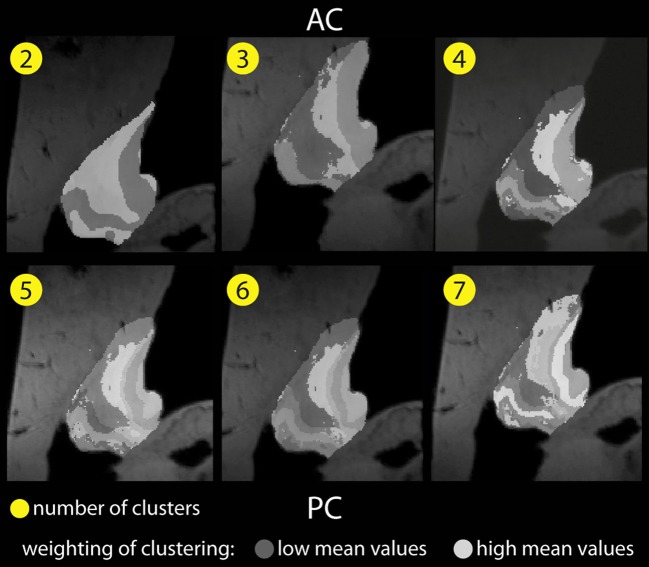
Habenular clustering based on image intensities in T_1_ (MP2RAGE) and T^*^_2_ (GRE) images of *ex vivo* data. *K* = 2, 3, 4, 5, 6, 7 clusters (from left above to right below).

Figure 5 shows color-coded images based on image intensities of all voxels within the habenular nucleus based on T_1_ and T^*^_2_ contrast for a given number of clusters. The weight of each cluster is represented by an arbitrary gray-white intensity scale where the lowest intensity is represented by the darkest shade and the brightest shade indicates high mean values. It depicts the difference between clusters when a Gaussian distribution of intensity in each cluster is assumed.

Anatomic details present in the *ex vivo* T_1_ and T^*^_2_ contrast MR data become more apparent, and show several compartments within the habenula. As the number of clusters increases, we find a distribution of intensities in the lateral habenula (LHB) that is more diverse than in medial habenula (MHB).

When 4 to 6 clusters are assumed, similar patterns are revealed and the clustering found appears to be less dependent on the number of clusters. The spatial configuration of the clusters found is also more consistent.

**Figure 6:**
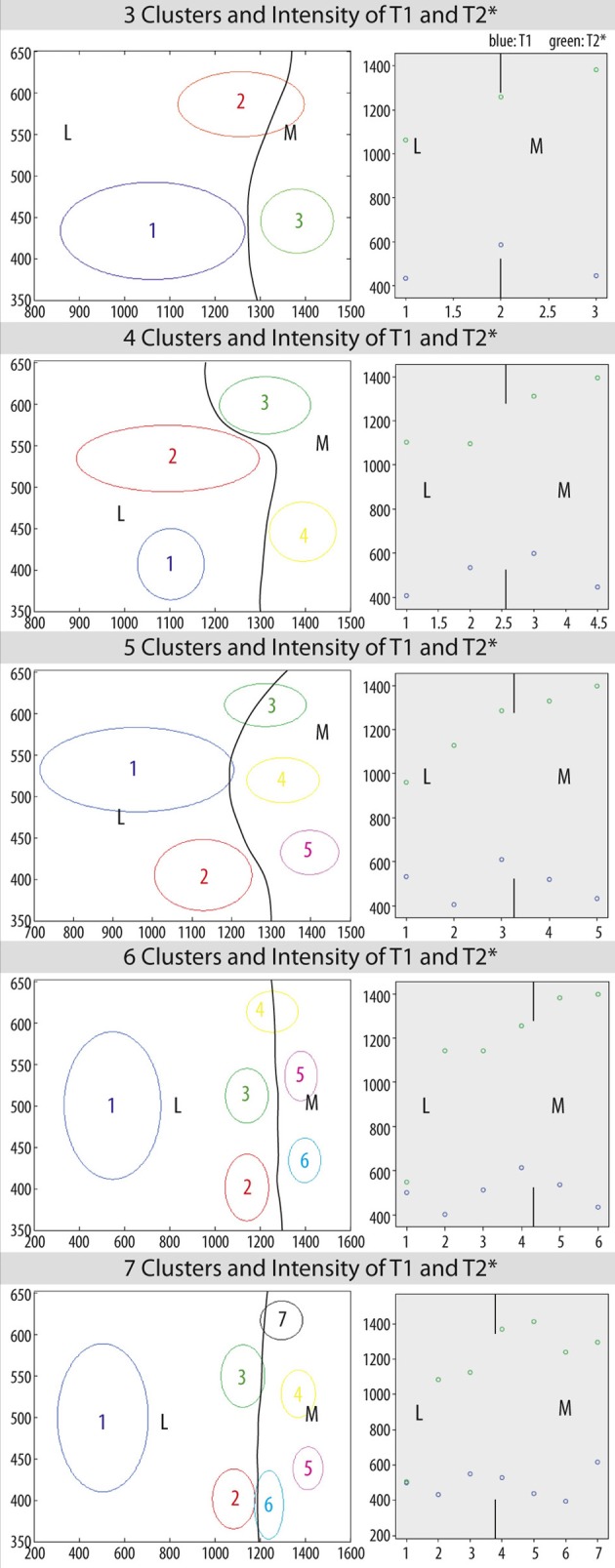
Mean intensity values of T1 and T2^*^ with standard error for each cluster (*k* = 3, 4, 5, 6, 7) as an ellipse (left) and distribution of mean intensity values for T1 (blue) and T2^*^ (green) (right) and subdivisions of habenula as marked by lines and indicated by M for medial habenula (MHB) and L for lateral habenula (LHB).

Figure 6 shows the mean intensity values of T_1_ and T^*^_2_ for each clustering with its standard error as an ellipse and the distribution of the mean intensities. Mean intensity values for each clustering reveal different subdivisions in lateral and medial habenula.

## Discussion

*Ex vivo* ultra-high resolution 7T MR imaging of the human habenula provides sufficient signal-to-noise and contrast-to-noise ratio to enable a clear visualization and identification of the lateral and medial nuclei of this important brain component.

Our data revealed heterogeneity of the lateral habenular (LHB) nuclei as suggested by Herkenham and Nauta (1977), Iwahori (1977), and Andres et al. (1999). A lateral and a medial part (l-LHB, m-LHB) can be clearly discriminated, and distinguished from the neighboring medial habenula (MHB).

Furthermore, advanced registration procedures and clustering methods showed subcomponents of lateral habenular nucleus as well as in medial habenula based on the contrast in quantitative maps of T1 and T2^*^. Between 4 and 6 clusters having different image intensities within the habenular nucleus can be observed, which distinguish between subdivisions of the lateral and medial habenular nuclei.

Lateral and medial habenula have previously been shown with histology in the human cadaver brain, and several subdivisions were shown in lateral and medial habenula in the animal brain. For the first time, we can observe subdivisions of lateral and medial habenula in the human brain. Using MRI, in particular, we can confirm heterogeneity of lateral habenula, which have previously been reported in the rat and cat brain.

Our findings are in line with previous findings based on structural and diffusion data of the human habenula that showed *in vivo* a subcompartment between medial and lateral habenula (Strotmann et al., 2013).

The foci of this study were high-field post-mortem MRI, the analysis of these images, and the potential for transfer of these findings for the interpretation of *in vivo* MRI scans. Because of the unique ability of MRI to visualize brain structures in three dimensions, *ex vivo* MRI is becoming increasingly important (e.g., Leuze et al., 2013). Future studies will include detailed correlation of these findings to histology.

Our *ex vivo* results should help to interpret *in vivo* scans of the habenula region, when the increasing well-understood reduction in relaxation parameters after fixation has been taken into account.

Use of a small-animal MR system will provide results with still higher spatial resolution. However, to facilitate comparison with future *in vivo* experiments, we performed this study using a 7T whole-body system, which enabled easy transfer of scanning parameters and sequences. The loss of SNR that might occur when using small tissue blocks and whole brain RF coils was avoided by the use of a custom-built miniature single loop coil. Similar image quality within reasonable scanning time and with (0.15 mm)^3^ resolution may be achievable *in vivo*, with optimized scan parameters and a 64-channel phased array RF receive coil. In addition, simultaneous slice excitation pulse techniques using only 2D phase encoding might be used for this purpose.

Our analytical approach reveals only the clustering of habenular tissue based on prior assumptions (*k* = 2–7 clusters). We compared clustering patterns and found that assuming 4 to 6 clusters gives similar findings, which appeared to be less dependent on the number of assumed clusters. Our analysis approach is based on this prior assumption, whereas a probabilistic clustering approach such as k-means clustering might be less prone to a potential bias, but harder to evaluate, especially due to the small sample size (*N* = 1).

The layered pattern we found is consistent with Iwahori (1977) description of the neuronal organization in the cat, where MHB was found to consist of small compactly arranged neurons of two types (piriform and fusiform shape), and LHB contained four different types of neurons (large, medium, small sized projections neurons, and small cells with short axons). Andres et al. (1999) described MHB in the rat brain as containing small, densely packed neurons, m-LHB with smaller cells and fewer myelinated fibers, l-LHB consisted of larger neurons and more myelinated fibers forming the lateral root of fasciculus retroflexus.

This finer parcellation of the habenula may be particularly useful in patients suffering from major depression where altered function and structure of the habenula, including cell loss and reduced volumes, has been reported (Ranft et al., 2010; Savitz et al., 2011). Inhibition of activity in the lateral habenula via pharmacology or deep brain stimulation (Sartorius et al., 2010; Winter et al., 2010) is thought to decrease the symptoms of depression toward remission.

Medial lateral habenula (m-LHB) is considered to be associated with the limbic system, and its lateral part (l-LHB) is thought to be linked to the motor system (Herkenham and Nauta, 1977).

The habenula's connectivity to the limbic emotional system and the motor system might be crucial to gain a deeper understanding of its role in psychiatric and neurological disorders. In major depression, the illness is described as a combination of different symptoms, mainly characterized by a negative mood which absorbs all energy and involves motor, vegetative, and cognitive symptoms. A sudden remission of treatment-resistant patients with major depression has been reported after DBS of lateral habenula by Sartorius et al. (2010). Therefore, future investigations and pharmacological research should target the lateral and medial part of lateral habenula and its role in this “core illness circuit” in major depression.

These findings may also help to guide fMRI studies of the habenula which explore the role of lateral habenula in healthy brain function and in disease.

### Conflict of interest statement

The authors declare that the research was conducted in the absence of any commercial or financial relationships that could be construed as a potential conflict of interest.
